# The Efficacy of PTH and Abaloparatide to Counteract Immobilization-Induced Osteopenia Is in General Similar

**DOI:** 10.3389/fendo.2020.588773

**Published:** 2020-10-09

**Authors:** Mikkel Bo Brent, Jesper Skovhus Thomsen, Annemarie Brüel

**Affiliations:** Department of Biomedicine, Aarhus University, Aarhus, Denmark

**Keywords:** μCT, bone formation, immobilization, PTH, abaloparatide

## Abstract

Immobilization results in a substantial bone loss and increased fracture risk. Powerful bone anabolic therapies are necessary to counteract the bone loss and reduce fracture risk during periods with immobilization. Intermittent parathyroid hormone 1−34 (PTH) (teriparatide) and PTH related peptide analog abaloparatide (ABL) are potent bone anabolic therapies acting through the same receptor, but induce different durations of signaling response. We investigated the efficacy of PTH or ABL in preventing immobilization-induced bone loss in rats in a direct mole-to-mole comparison. Immobilization was achieved by injecting botulinum toxin type A (BTX) into the right hindlimb musculature. Sixty 14-week-old female Wistar rats were allocated to the following groups: Baseline, Control, BTX, BTX + PTH (80 μg/kg/day), and BTX + ABL (77 μg/kg/day). Immobilization resulted in a substantial and significant reduction in bone mineral density (aBMD), metaphyseal and epiphyseal trabecular bone volume fraction (BV/TV) and trabecular thickness (Tb.Th), metaphyseal trabecular number (Tb.N), and femoral neck bone strength. Both PTH and ABL prevented the immobilization-induced decrease in aBMD, metaphyseal and epiphyseal Tb.Th, and metaphyseal Tb.N. In addition, PTH rescued the reduction in metaphyseal BV/TV and femoral neck strength, while ABL did not. However, the effect of PTH and ABL did not differ significantly for serum calcium, aBMD, metaphyseal, and epiphyseal BV/TV, Tb.Th, or Tb.N. In conclusion, in a mole-to-mole comparison the efficacy of PTH and ABL is similar in counteracting immobilization-induced reduction in bone mineral density, deterioration in trabecular microarchitecture, and decrease in bone strength.

## Introduction

Bone remodeling is a life-long ongoing process of adaptation of the skeleton to internal and external stimuli (1). Mechanical loading plays a fundamental role in maintaining a healthy balance between bone resorption and bone formation (2). Reduced weight-bearing and skeletal muscle load as a result of immobilization, such as strict bedrest or spinal cord injury, accelerates bone loss due to increased osteoclastic bone resorption (3–5). Potent bone anabolic therapies are needed in order to counteract or restore the loss of bone due to immobilization.

Parathyroid hormone (PTH) (1–34) or teriparatide is a potent bone anabolic drug, when given intermittently, approved for treatment of osteoporosis in both women and men (6–9). PTH increases bone turnover mediated by direct stimulation of osteoblasts and by indirect stimulation of osteoclasts (10). In contrast to continuously elevated PTH (11), intermittent administration of PTH results in more bone formation than bone resorption (12, 13). Abaloparatide (ABL) is a synthetic polypeptide with 41% sequence homology to PTH and 76% homology to parathyroid hormone-related protein (PTHrP), which increases net bone formation when administered intermittently (14).

**Figure 1:**
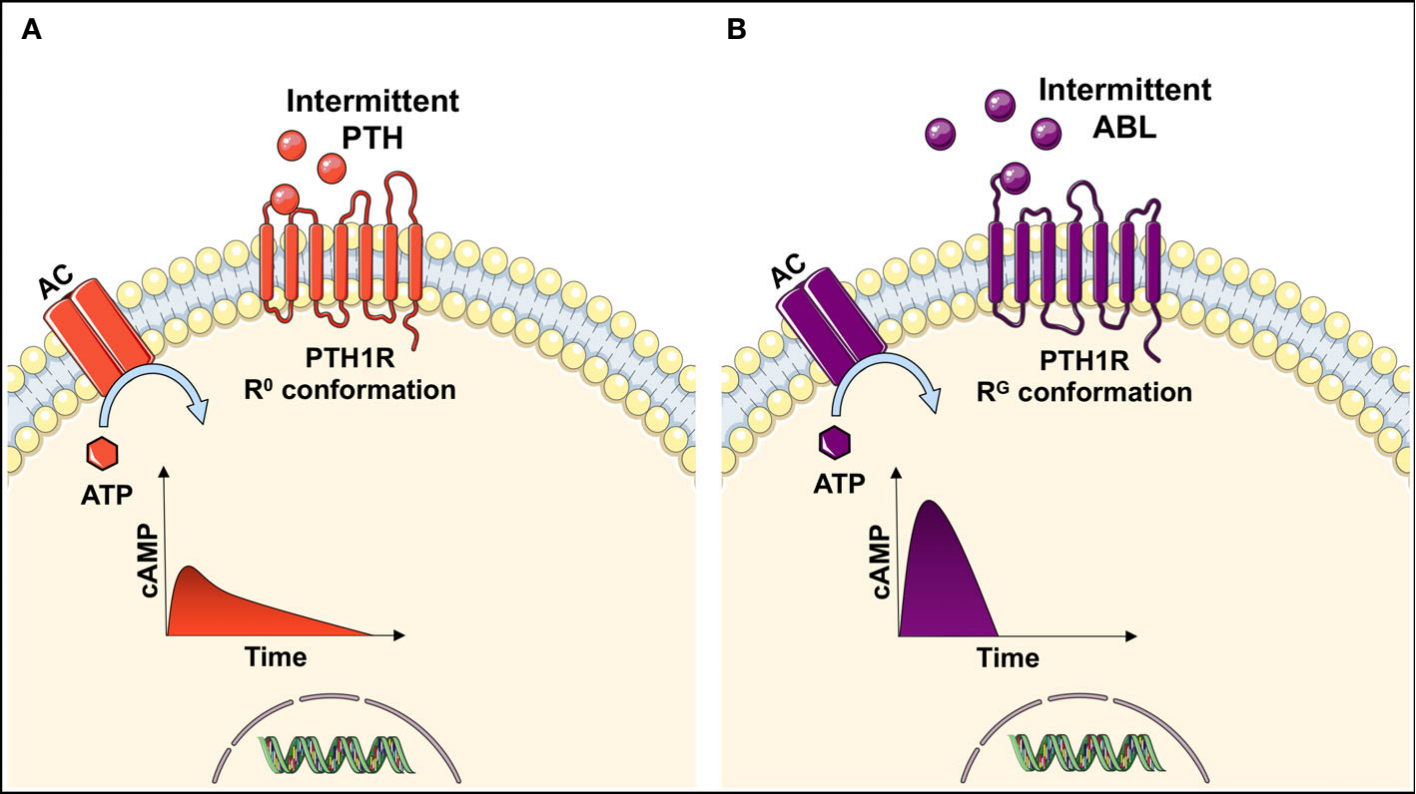
Intracellular signaling response after parathyroid hormone (PTH) **(A)** or abaloparatide (ABL) **(B)** binds to the parathyroid hormone 1 receptor (PTH1R) on osteoblasts. When PTH binds to the R0 conformation of the receptor the adenyl cyclase (AC) converts adenosine triphosphate (ATP) to cyclic adenosine monophosphate (cAMP), thus resulting in a prolonged increase in cAMP. In contrast to abaloparatide, which is more selective in favor of binding the RG conformation of PTH1R and thereby inducing a more temporal signaling response by a short and rapid increase in cAMP (9, 19, 20).

The parathyroid hormone 1 receptor (PTH1R) is a G-protein coupled receptor expressed on osteoblasts that functions as the target receptor for PTH and PTHrP. When PTH or PTHrP binds to PTH1R, an intracellular signaling cascade is initiated involving multiple downstream pathways ultimately resulting in increased osteoblast differentiation and activity and reduced osteoblast apoptosis (15–17). However, the PTH1R exits in two distinct conformations, R^0^ and R^G^ (18). Ligand binding to the R^0^ conformation results in a prolonged signaling response, while ligands binding to the R^G^ conformation induce a more temporal and rapid signaling response (19, 20). Several *in vivo* and *in vitro* studies have reported PTH to be selective toward the R^0^ receptor conformation, whereas PTHrP preferentially binds to the R^G^ receptor conformation (19–21). A simplified intracellular signaling response of PTH and PTHrP is shown in **Figure 1**.

The selectivity of the receptor conformation has been exploited to develop the drug abaloparatide, which preferentially binds to the R^G^ conformation of PTH1R, thereby inducing a more transient and rapid signaling response resulting in reduced osteoclastic bone resorption. The importance of signaling response duration is underlined by clinical studies showing that intermittent PTH has a higher risk of hypercalcemia (6) than abaloparatide (22, 23). Hypercalcemia is potentially life-threating and can lead to lethargy, confusion, shortened QT-interval and cardiac arrhythmias (24, 25).

A comparison of several different dosages of PTH and abaloparatide in a 4:1 ratio have previously shown that both drugs improve fracture healing in mice (26). However, no study has yet investigated PTH and abaloparatide in a direct mole-to-mole comparison in prevention of immobilization-induced osteopenia.

The objective of the present study was to make the first direct mole-to-mole comparison of PTH and abaloparatide in hindlimb-immobilized rats.

## Materials And Methods

### Animals and Study Design

Sixty 14-week-old female Wistar rats were obtained from Janvier Labs (Le Genest-Saint-Isle, France) with a mean body weight (BW) of 276 ± 12 g and housed at 20°C with a 12:12 h light/dark cycle. The animals had free access to tap water and standard rat chow (1324 maintenance diet for rats and mice; Altromin, Lage, Germany). Seven days prior to study start the animals were allocated to five groups (*n* = 12/group) based on their BW: baseline (Base), control (Ctrl), Botulinum toxin A (BTX), BTX + PTH, and BTX + ABL. Animals in the Base group were sacrificed at study start to determine age-dependent skeletal development during the study. At study start, 4 IU of botulinum toxin type A (Botox, Allergan, Dublin, Ireland) were injected into the right quadriceps, hamstring, and calf musculature, thus inducing chemical denervation and disuse, whereas animals allocated to Ctrl were injected with saline as previously described (12). Human PTH (1–34) (H-4835, Bachem, Bubendorf, Switzerland) (80 μg/kg/day) and ABL (H-8334, Bachem, Bubendorf, Switzerland) (77 μg/kg/day) was administered as s.c. injections 5 days a week to animals in the BTX + PTH and BTX + ABL groups, respectively. Consequently, animals in non-treatment groups (Ctrl and BTX) received injections with saline s.c. five times a week. The dosage of PTH [relative molecular mass (M_r_) = 4117.77 Da] is similar to that used in previous studies (12, 27, 28), and the dosage of ABL (M_r_ = 3960.64 Da) allows a mole-to-mole comparison between the two drugs.

In order to asses bone formation both early and late in the experiment, the animals were injected s.c. with calcein (5 mg/kg, C0875, Sigma-Aldrich, St. Louis, MO, USA) 0 and 1 week after study start and alizarin (20 mg/kg, A3882, Sigma-Aldrich, St. Louis, MO, USA) 2 and 3 weeks after study start.

The physiological effect of BTX on voluntary movement and muscle contraction of the injected hindlimb was assessed with the gait ability score described by Warner et al. (29). After four weeks, the animals were killed under general anesthesia with isoflurane (Attane Vet; ScanVet, Fredensborg, Denmark) and an overdose of 200 mg/kg of pentobarbital i.p. (Mebumal, SAD, Copenhagen, Denmark). One rat from the BTX + ABL group was euthanized prematurely because of failure to thrive.

After the animals were killed, the right hindlimbs were removed and femora, and tibiae were isolated in a standardized manner. The femora were stored in Ringer’s solution at −20°C, and the tibiae were immersion-fixed in 0.1 M sodium phosphate-buffered formaldehyde (4% formaldehyde, pH 7.0) for 48 h and then stored in 70% ethanol. All procedures were approved by the Danish Animal Experiment Inspectorate (2018-15-0201-01436) and followed the ARRIVE guidelines for reporting *in vivo* experiments (30).

### Serum Analyses

Blood was collected from the inferior vena cava right before the animals were killed. The blood was stored in Eppendorf tubes on ice for 15 min before centrifugation at 1100 g for 10 min at 4°C in order to separate serum. Serum was stored at −80°C before the calcium ion concentrations was determined using a colorimetric assay kit (MAK022, Sigma-Aldrich, St. Louis, MO, USA) in accordance with the manufacture’s guidelines. Calcium ion content in serum was compared with a standard curve generated by measuring a series of samples containing different known concentrations of ionized calcium.

### Dual-Energy X-Ray Absorptiometry

Remaining soft tissue was carefully removed before scanning the right femora in a dual-energy x-ray absorptiometry (DXA) scanner (Sabre XL, Nordland Stratec, Pfortzheim, Germany). A scan speed of 10 mm/s and an isotropic pixel size of 0.5 mm was used. Quality assurance was performed by scanning the two solid-state phantoms provided with the scanner according to the manufacture’s guidelines.

### Micro–Computed Tomography

The right femoral mid-diaphysis, the distal femoral metaphysis and epiphysis, and corpus of the L5 vertebra were scanned in a desktop micro–computed tomography (μCT) scanner (Scanco μCT 35, Scanco Medical AG, Brüttiselen, Switzerland). Three different bone sites were scanned, since immobilization-induced bone loss previously has been reported to be site-specific in rodents (31). All scans were performed in high-resolution mode (1000 projections/180°) with an isotropic voxel size of 10 μm, an X-ray tube voltage of 70 kVp and current of 114 μA, and an integration time of 800 ms. In addition, beam-hardening effects were reduced using a 0.5-mm aluminum filter.

**Figure 2:**
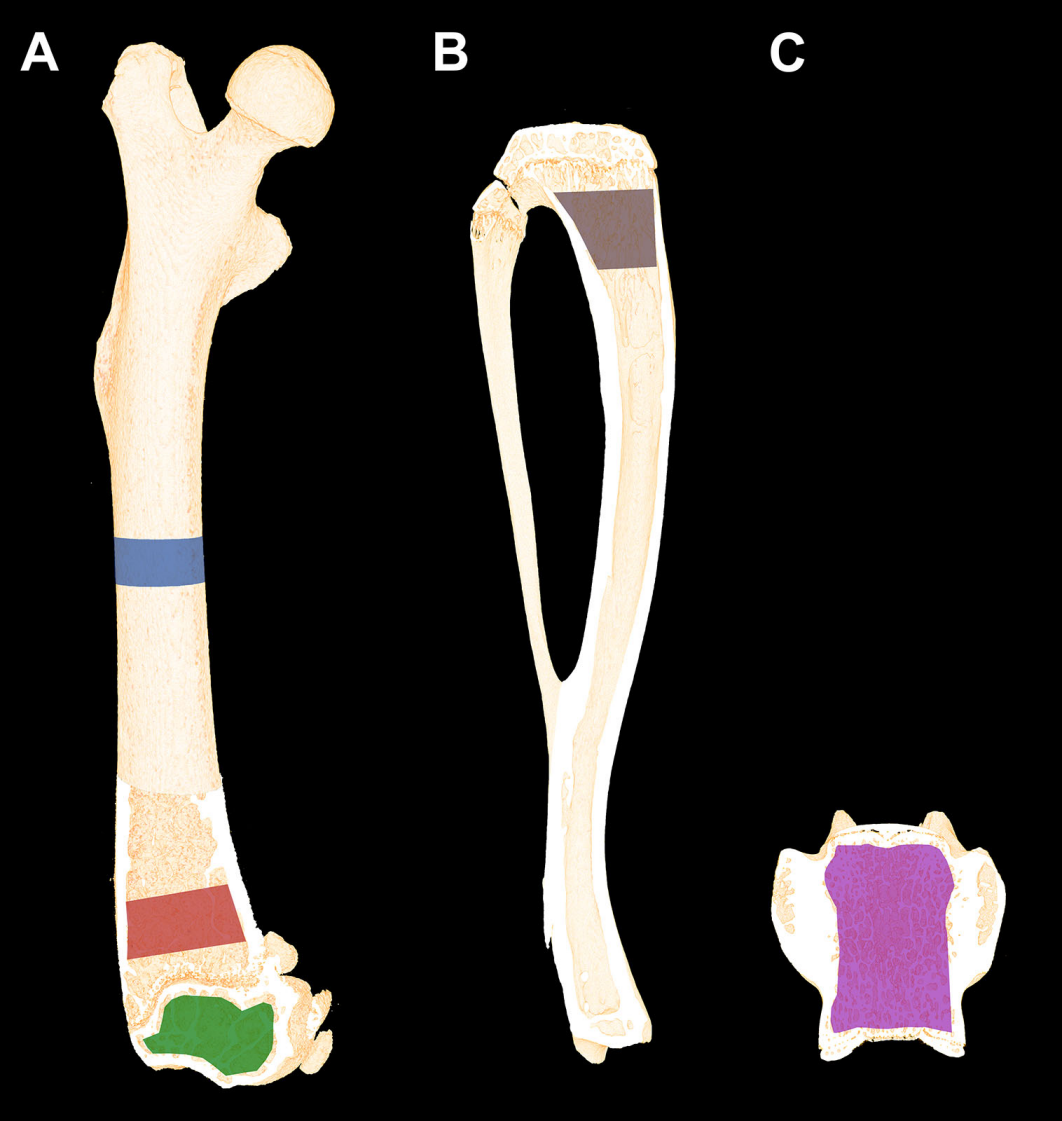
**(A)** Schematic illustration of the volumes of interest (VOIs) scanned with μCT. Blue: Femoral mid-diaphyseal VOI. Red: Distal femoral metaphyseal VOI. Green: Distal femoral epiphyseal VOI. **(B)** Region of interest (ROI) of the proximal tibial metaphysis analyzed using dynamic bone histomorphometry and bone cell counting. Dynamic bone histomorphometry was also performed at the femoral mid-diaphysis. **(C)** The VOI used to analyze L5. Dimensions are not to scale.

Volumes of interest (VOIs) were drawn semi-manually using the software provided with the μCT scanner (Evaluation v. 6.5, Scanco Medical AG, Brüttiselen, Switzerland) as previously described (12). In brief, the mid-diaphysis was analyzed using a 2320-μm high VOI including cortical bone only (**Figure 2A**). The distal femoral metaphysis was analyzed using a 2200-μm high VOI starting 1500 μm above the most distal part of the growth plate in order to exclude primary spongiosa. The distal femoral epiphysis was analyzed using a 1720-μm high VOI starting, where the lateral and medial condyle fused to one coherent structure and ended where the growth plate first appeared (**Figure 2A**). Corpus of the L5 vertebra was analyzed using a VOI containing the whole trabecular network from upper to lower growth plate (**Figure 2C**). The 3D data sets were low-pass filtered using a Gaussian filter (σ = 0.8, support = 1) and segmented with a fixed threshold filter of 532 mg HA/cm^3^. The assessment of the bone microstructure using μCT was performed in accordance with the current guidelines (32).

### Mechanical Testing

The load to fracture of the femoral mid-diaphysis, femoral neck, and distal femoral metaphysis were determined using a materials-testing machine (5566; Instron, High Wycombe, UK) as previously described in detail (12). In brief: Vertical load was applied with a constant deflection rate of 2 mm/min until a complete fracture was achieved. During the mechanical testing, load-deformation data were recorded using Merlin (version 3.21, Instron) and subsequently analyzed using a custom-made computer program.

### Dynamic Histomorphometry, Osteoblast Covered Surfaces, and Osteoclast Covered Surfaces

#### Cortical Bone

After mechanical testing, an approximately 200-μm-thick cross-sectional section of the femoral mid-diaphysis was sawed from the proximal part with a diamond precision-parallel saw (Exakt Apparatebau, Norderstedt, Germany) and mounted with Pertex on glass slides. Fluorochrome labels (calcein, and alizarin) were counted using a light microscope specially equipped for fluorescence (Nikon Eclipse i80, Tokyo, Japan). Live images of the slides were transmitted to a computer equipped with newCAST (v. 2019.07.3, Visiopharm, Hørsholm, Denmark), where a 24-arm grid was superimposed on the live images. Only fluorochrome labels intersecting with one of the positions of the 24-arm grid on the periosteal and endosteal bone surfaces were counted. The distance between two calcein or between two alizarin labels was determined using the integrated measuring tool in the newCAST software. Mineralizing surfaces (MS/BS), mineral appositional rate (MAR), and bone formation rate (BFR/BS) were calculated as previously described at a final magnification of ×1132 (12).

#### Trabecular Bone

**Figure 3:**
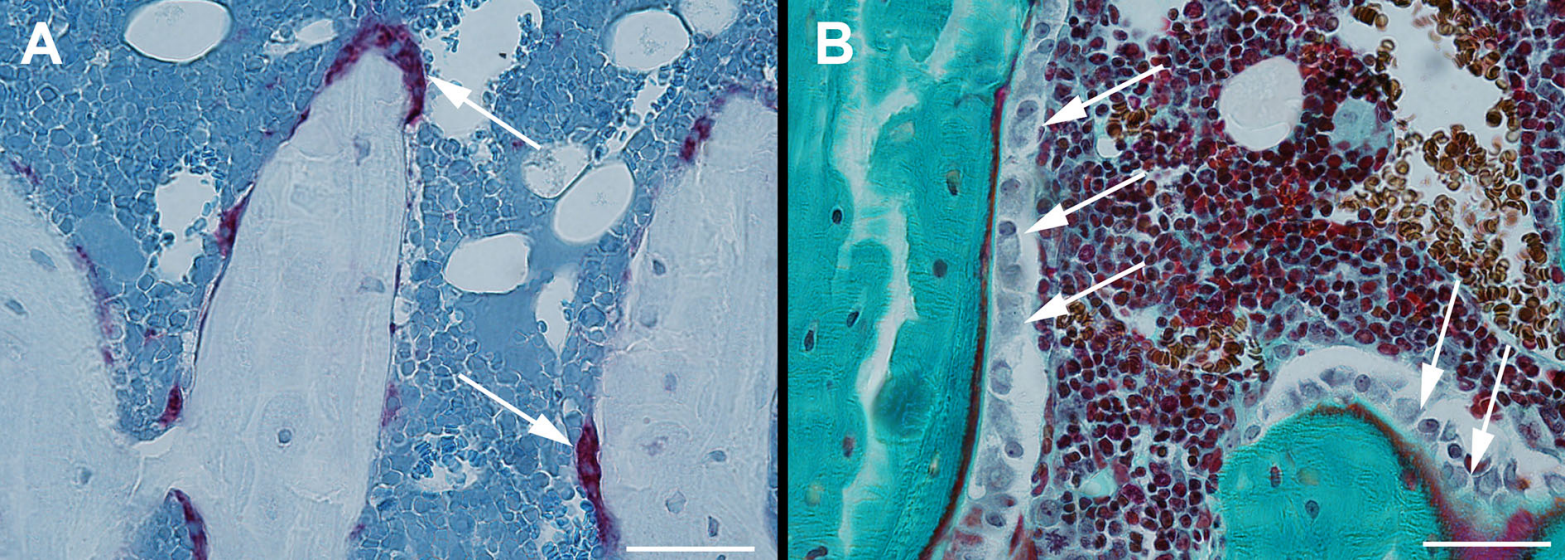
Sagittal sections of the proximal tibia. Section stained for tartrate-resistant acid phosphatase (TRAP) and counterstained with aniline blue for osteoclast counting **(A)** and Masson-Goldner trichrome stained for osteoblast and osteoid counting **(B)**. Bar = 50 μm.

The proximal tibiae were embedded undecalcified in methyl methacrylate (MMA) and sagittal 7-μm-thick sections were cut using a microtome (Jung RM2065; Leica Instruments, Nussloch, Germany). The sections were either left unstained for dynamic bone histomorphometry (**Figure 3A**), stained for tartrate-resistant acid phosphatase (TRAP), and counterstained with aniline blue for osteoclast counting (**Figures 2B**, **3B**) or Masson-Goldner trichrome stained for osteoblast and osteoid counting (**Figures 2B**, **3C**). MS/BS, MAR, BFR/BS, osteoid covered surfaces (OS/BS), osteoclast covered surfaces (Oc.S/BS), and osteoblast covered surfaces (Ob.S/BS) were determined in a 3000-μm-long ROI starting 1500 μm below the growth plate, thus including trabecular bone only. Randomly rotated line grids were superimposed on the live images using the newCAST software at a final magnification of ×1132.

The histological assessments were all performed by the same operator who was blinded for the group distribution. If double labels were absent, then an imputed value of zero was used for MAR and BFR/BS. The assessment of bone histomorphometry and usage of related nomenclature was performed in accordance with the current recommendations by the ASBMR Histomorphometry Nomenclature Committee (33).

### Statistics

Differences between groups were analyzed using a parametric one-way ANOVA followed by a post-hoc Holm-Sidak test, if the data were normally distributed, otherwise a non-parametric one-way ANOVA on ranks followed by a post-hoc Dunn’s test was used. The one-way ANOVA included the groups Ctrl, BTX, BTX + PTH, and BTX + ABL. Data are presented as means ± SD and results were defined as statistically significant if the two-tailed *p*-value was less than 0.05. The statistical analyses were performed in GraphPad Prism 8.2.1 (Systat Software, Chicago, IL, USA).

An *a priori* sample size calculation (power = 0.8) showed, that it is possible to demonstrate a difference of 12% in mechanical strength of the mid-femur between groups with 12 animals in each group (28).

## Results

### Animals

**Figure 4:**
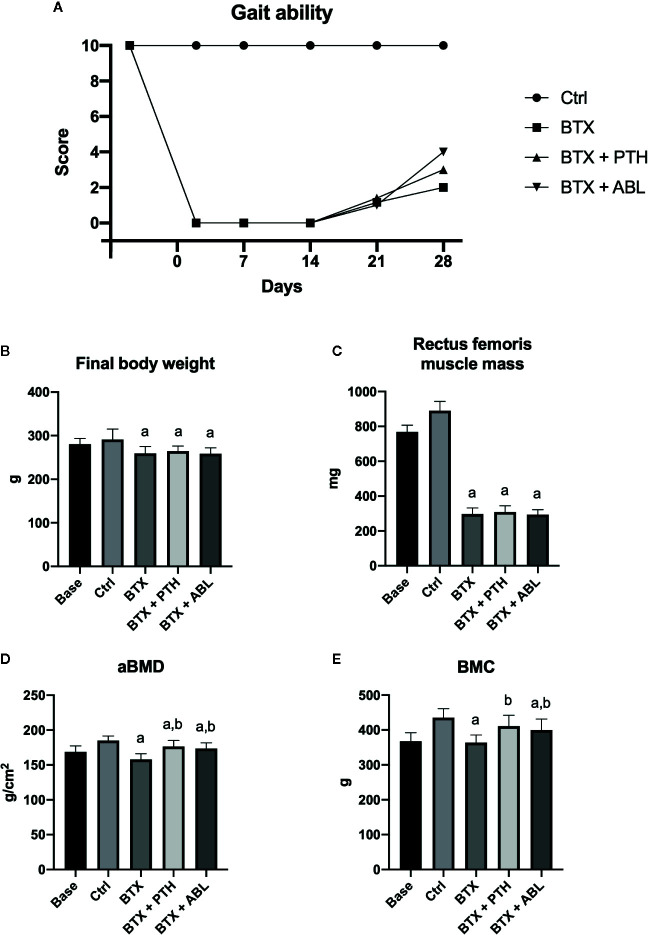
**(A)** Gait ability score after right hindlimb injections with BTX. A gait ability score of 0 indicates profound paresis, whereas a score of 10 indicates normal gait ability. **(B)** Final body weight at study end. **(C)** Wet weight of rectus femoris muscle. **(D)** Femoral aBMD, bone mineral density. **(E)** Femoral BMC, bone mineral content. Data in **(B–E)** are presented as means ± SD. a: *p* < 0.05 vs. Ctrl. b: *p* < 0.05 vs. BTX.

Immobilization resulted in significantly lower bodyweight (−11%) and a decreased rectus femoris muscle weight (−67%) compared with Ctrl at the end of the study. Neither the body weight nor the rectus femoris muscle mass was affected by treatment with PTH or ABL (**Table 1** and **Figure 4**). The gait ability rapidly declined after injections with BTX, and the reduced gait ability lasted throughout the study. However, a slow increase in gait ability was observed after the first week and onwards, but the gait ability never fully recovered (**Figure 4**).

**Table 1:** Number of animals, BW at study start, BW at study end, femoral length, rectus femoris muscles mass, and ionized calcium in serum of rats injected with BTX into the right hindlimb and treated with PTH or ABL.

	Base	Ctrl	BTX	BTX + PTH	BTX + ABL
Number of animals	12	11	12	12	12
BW start (g)	280 ± 13	279 ± 12	279 ± 10	274 ± 10	271 ± 15
Femoral length (mm)	34.0 ± 0.5	35.6 ± 0.5	35.3 ± 0.7	35.7 ± 0.8	35.4 ± 0.7
Serum calcium (mmol/l)	2.81 ± 0.54	3.24 ± 0.33	3.08 ± 0.35	2.73 ± 0.31^a^	2.81 ± 0.34

Ionized calcium in serum was determined from eight animals per group. Data are presented as mean ± SD. ^a^p < 0.05 vs. Ctrl.

### Serum Analysis

The concentration of ionized calcium in serum did not differ between animals treated with PTH and ABL (**Table 1**).

### DXA

Animals in the BTX group had significantly reduced aBMD (−15%) and BMC (−17%) compared with Ctrl animals. Both treatment with PTH and ABL increased aBMD (+11 and +10%) and BMC (+13 and +10%) compared with BTX, respectively. No significant difference was found between PTH and ABL for either aBMD or BMC (**Figure 4**).

### μCT

#### Femoral Mid-Diaphysis

Immobilization resulted in significantly lower B.Ar (−8%) compared to non-immobilized animals, whereas no effect of immobilization was found for T.Ar, M.Ar, BA/TA, or Ct.Th. Treatment with either PTH or ABL did not counteract the immobilization-induced decrease in B.Ar (**Table 2**).

**Table 2:** Microstructural properties of the right femoral mid-diaphysis from rats with the right hindlimb immobilized with BTX and treated with PTH or ABL.

	Base	Ctrl	BTX	BTX + PTH	BTX + ABL
B.Ar (mm^2^)	5.44 ± 0.28	6.12 ± 0.29	5.60 ± 0.32^a^	5.84 ± 0.37	5.82 ± 0.46
T.Ar (mm^2^)	8.93 ± 0.71	9.85 ± 0.62	9.19 ± 0.72	9.40 ± 0.67	9.32 ± 0.91
M.Ar (mm^2^)	3.49 ± 0.50	3.73 ± 0.44	3.60 ± 0.48	3.56 ± 0.44	3.50 ± 0.52
BA/TA (%)	61.1 ± 2.78	62.3 ± 2.56	61.0 ± 2.55	62.2 ± 2.72	62.6 ± 2.60
Ct.Th (mm)	0.56 ± 0.03	0.62 ± 0.05	0.59 ± 0.02	0.59 ± 0.04	0.62 ± 0.04

B.Ar, bone area; T.Ar, tissue area; M.Ar, marrow area; BA/TA, bone area/tissue area; Ct.Th, cortical thickness. Data are presented as mean ± SD. ^a^p < 0.05 vs. Ctrl.

#### Distal Femoral Metaphysis

**Figure 5:**
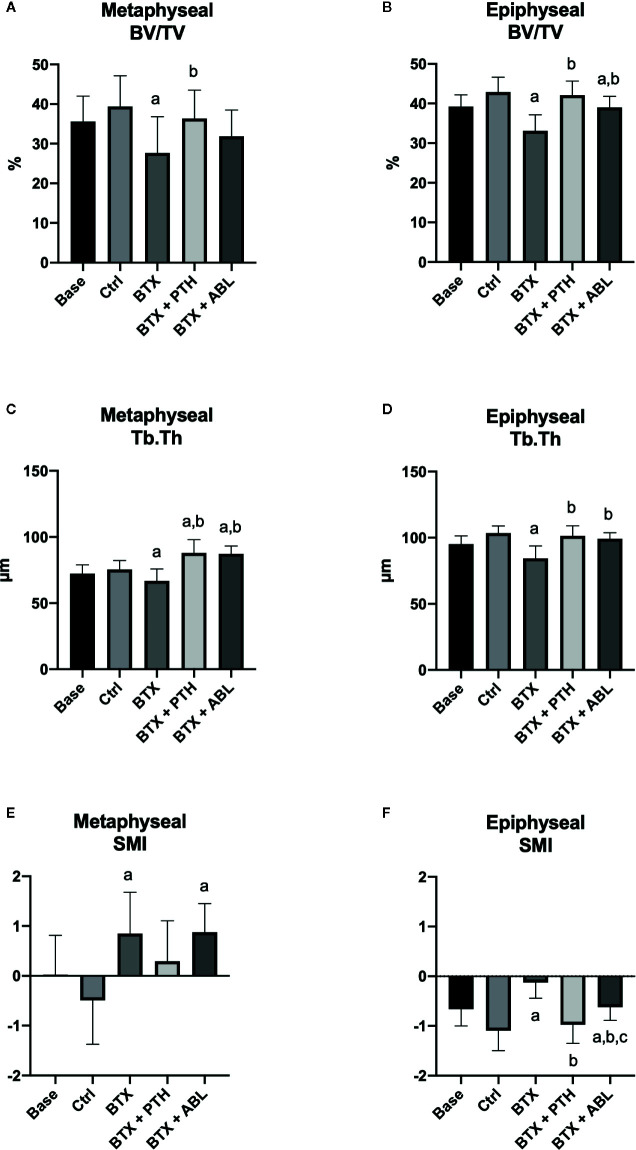
Distal femoral metaphyseal and epiphyseal trabecular bone parameters determined by μCT from rats with the right hindlimb immobilized with BTX and treated with PTH or ABL. **(A**, **B)** BV/TV, bone volume/tissue volume; **(C**, **D)** Tb.Th, trabecular thickness; **(E**, **F)** SMI, structure model index. Data are presented as mean ± SD. a: p < 0.05 vs. Ctrl. b: p < 0.05 vs. BTX. c: p < 0.05 vs. BTX + PTH.

BTX significantly decreased BV/TV (−30%), Tb.Th (−12%), Tb.N (−15%), and vBMD (−24%) and increased SMI (+274%). Both PTH and ABL completely prevented the disuse-induced loss of Tb.Th reaching a level significantly above that of Ctrl (+16%, for both treatment regimens). Only treatment with PTH significantly increased BV/TV (+32%) compared with BTX, while treatment with ABL did not. Treatment with PTH or ABL did not affect Tb.N, vBMD, or SMI compared with non-treated BTX animals. Finally, in general PTH and ABL did not differ in their ability to prevent the disuse-induced deterioration of bone micro-architecture at the distal femoral metaphysis (**Table 3** and **Figure 5**).

**Table 3:** Microstructural properties of the right femoral epiphysis and metaphysis from rats with the right hindlimb immobilized with BTX and treated with PTH or ABL.

	Base	Ctrl	BTX	BTX + PTH	BTX + ABL
Femoral metaphysis					
Tb.N (mm^−1^)	5.65 ± 0.75	5.85 ± 0.80	5.00 ± 0.74^a^	4.84 ± 0.50^a^	4.45 ± 0.53^a^
Tb.Sp (μm)	162 ± 24.9	155 ± 27.1	186 ± 38.3	179 ± 25.9	199 ± 34.5^a^
CD (mm^−3^)	231 ± 51.0	231 ± 51.0	206 ± 50.0	167 ± 29.4^a^	142 ± 25.9^a,b^
vBMD (mg/cm^3^)	421 ± 56.9	459 ± 70.3	350 ± 90.3^a^	433 ± 64.1^a^	388 ± 63.9
TMD (mg/cm^3^)	895 ± 9.48	912 ± 16.5	901 ± 16.9	900 ± 12.1	884 ± 15.4^a^
Femoral epiphysis					
Tb.N (mm^−1^)	3.90 ± 0.29	3.93 ± 0.28	3.93 ± 0.70	3.96 ± 0.20	3.78 ± 0.19
Tb.Sp (μm)	230 ± 17.8	227 ± 22.5	238 ± 31.0	226 ± 13.7	239 ± 16.4
CD (mm^−3^)	102 ± 16.9	87.7 ± 12.0	113 ± 52.9	91.6 ± 6.75	89.5 ± 10.4
vBMD (mg/cm^3^)	446 ± 28.0	485 ± 36.3	392 ± 41.7^a^	480 ± 35.2^b^	449 ± 27.7^b^
TMD (mg/cm^3^)	890 ± 9.88	911 ± 11.0	891 ± 14.7^a^	907 ± 11.2^b^	895 ± 7.10^a,c^

Tb.N, trabecular number; Tb.Sp, trabecular spacing; CD, connectivity density; vBMD, volumetric bone mineral density; TMD, tissue mineral density. Data are presented as mean ± SD. ^a^p < 0.05 vs. Ctrl. ^b^p < 0.05 vs. BTX. ^c^p < 0.05 vs. BTX + PTH.

#### Distal Femoral Epiphysis

**Figure 6:**
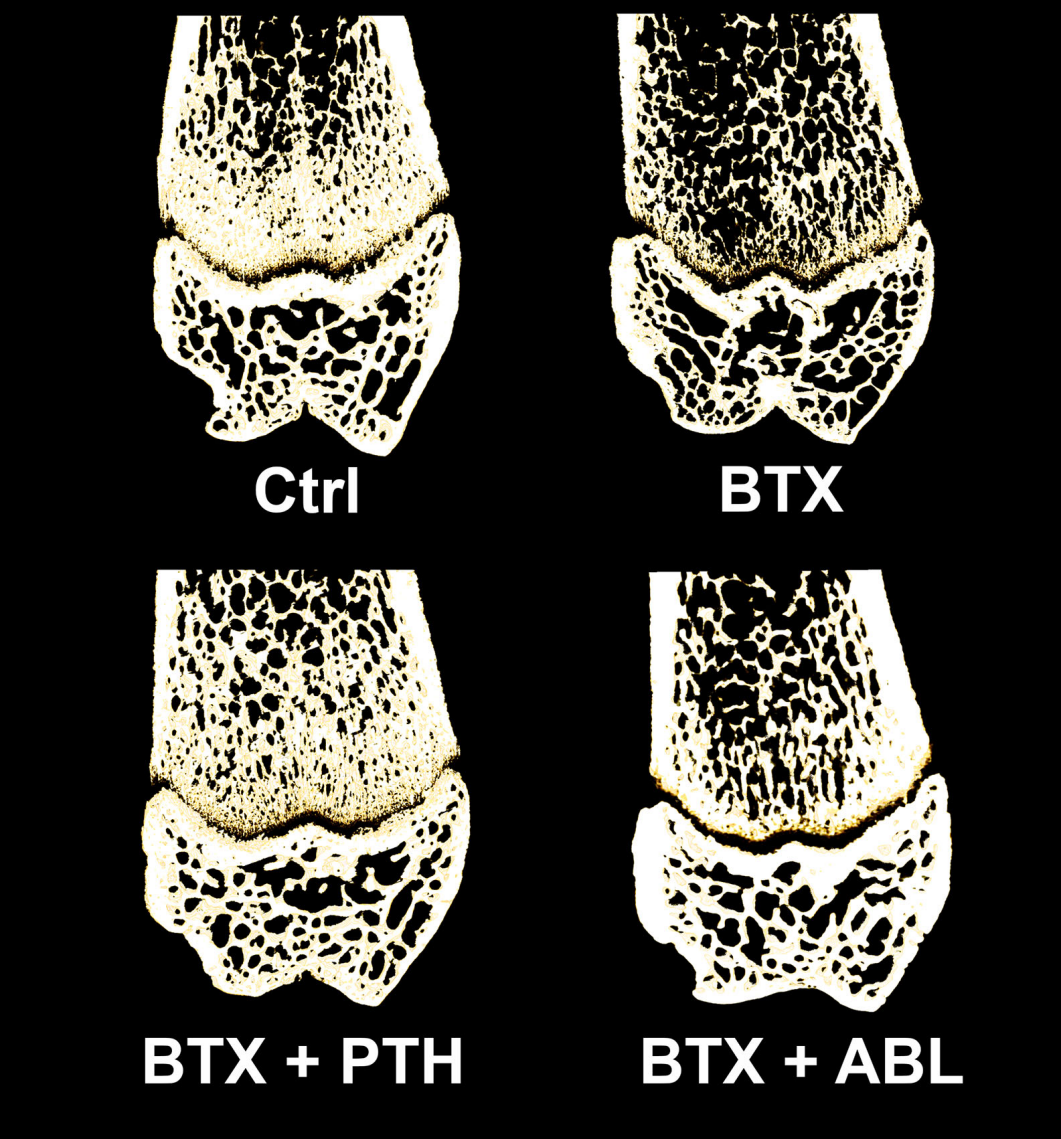
Representative three-dimensional images of a 100-μm-thick slice through the distal femoral metaphysis and epiphysis from rats with the right hindlimb immobilized with BTX and treated with PTH or ABL. Metaphyseal and epiphyseal BV/TV values for the depicted bone samples; Ctrl: 38% and 44%, BTX: 24% and 33%, BTX + PTH: 34% and 41%, BTX + ABL: 31% and 39%, respectively.

Immobilization significantly decreased BV/TV (−23%), Tb.Th (−19%), vBMD (−19%), and TMD (−2%) and increased SMI (+89%). Both treatment with PTH and ABL completely counteracted the disuse-induced trabecular thinning (+20 and +18%) compared to BTX. The decrease in BV/TV was prevented by PTH (+27% compared with BTX) reaching the level of Ctrl animals. However, the decrease in BV/TV was only partly prevented by ABL (+18% compared with BTX), to a level significantly below Ctrl. Furthermore, vBMD was significantly elevated compared with BTX for both treatment with PTH (+22%) and ABL (+15%), while TMD (+2%) was only increased in animals treated with PTH. The immobilization-induced increase in SMI was attenuated by both PTH and ABL. The effect of PTH and ABL differed significantly for TMD (−1%) and SMI (+37%), while the effect of PTH and ABL was similar for all the other microarchitectural parameters (**Table 3** and **Figures 5**, **6**).

#### Vertebra

BTX significantly decreased BV/TV (−8%) and Tb.Th (−6%), while no effect was found on Tb.N, Tb.Sp, CD, vBMD, TMD, and SMI. Both treatment with PTH and ABL completely counteracted the disuse-induced bone loss of BV/TV (+39 and +34%) and Tb.Th (+34 and +36%) to a level above Ctrl. In addition, treatment with PTH or ABL significantly increased vBMD (+22 and +17%) and decreased CD (−11 and −17%) and SMI (−103 and −61%) compared to Ctrl, respectively (**Table 4**).

**Table 4:** Microstructural properties of L5 from rats with the right hindlimb immobilized with BTX and treated with PTH or ABL.

	Base	Ctrl	BTX	BTX + PTH	BTX + ABL
BV/TV (%)	37.7 ± 2.71	37.4 ± 3.75	34.5 ± 2.48^a^	47.9 ± 4.06^b^	46.1 ± 3.82^a,b^
Tb.Th (μm)	75.6 ± 4.57	78.1 ± 3.08	73.4 ± 3.26^a^	98.1 ± 4.99^a,b^	100 ± 4.59^a,b^
Tb.N (mm^−1^)	4.74 ± 0.25	4.57 ± 0.35	4.47 ± 0.22	4.75 ± 0.31	4.53 ± 0.28
Tb.Sp (μm)	193 ± 12.3	200 ± 14.7	205 ± 11.2	180 ± 14.7^a,b^	193 ± 18.1
CD (mm^−3^)	155 ± 21.8	127 ± 15.7	133 ± 16.0	113 ± 11.1^a,b^	105 ± 9.91^a,b^
vBMD (mg/cm^3^)	426 ± 28.4	428 ± 40.1	404 ± 25.0	522 ± 41.5^a,b^	499 ± 41.9^a,b^
TMD (mg/cm^3^)	894 ± 9.20	906 ± 15.3	898 ± 9.20	912 ± 11.9^b^	905 ± 13.4
SMI	−0.76 ± 0.35	−0.77 ± 0.49	−0.45 ± 0.31	−1.56 ± 0.55^a,b^	−1.24 ± 0.46^a,b^

BV/TV, bone volume/tissue volume; Tb.Th, trabecular thickness; Tb.N, trabecular number; Tb.Sp, trabecular spacing; CD, connectivity density; vBMD, volumetric bone mineral density; TMD, tissue mineral density; SMI, structure model index. Data are presented as mean ± SD. ^a^p < 0.05 vs. Ctrl. ^b^p < 0.05 vs. BTX.

### Mechanical Testing

**Figure 7:**
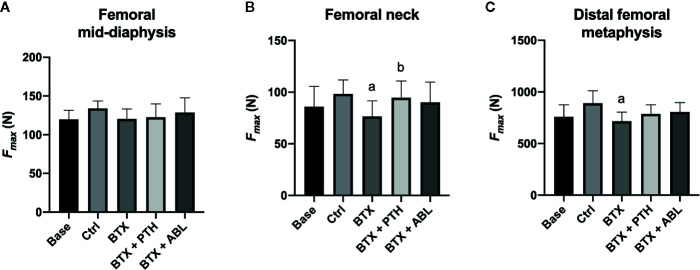
Fracture strength (*F*_max_) of the femoral mid-diaphysis **(A)**, femoral neck **(B)**, and distal femoral metaphysis **(C)** of rats with the right hindlimb immobilized with BTX and treated with PTH or ABL. Data are presented as mean ± SD. a: *p* < 0.05 vs. Ctrl. b: *p* < 0.05 vs. BTX.

Disuse significantly decreased femoral neck strength (−22%) and distal femoral metaphyseal strength (−20%), while no significant effect was found at the femoral mid-diaphysis. Treatment with PTH just barely (*p* = 0.047) increased femoral neck strength by 23% compared with BTX, while ABL did not (**Figure 7**). The effect of PTH and ABL did not differ for distal femoral metaphyseal and femoral mid-diaphyseal bone strength.

### Dynamic Bone Histomorphometry, Osteoblast, and Osteoclast Covered Bone Surfaces

#### Femoral Mid-Diaphysis: Endocortical Surface

No significant effect of disuse was found at the endocortical bone surface. Both PTH and ABL significantly increased MS/BS (+178 and +168%) compared with Ctrl, while PTH also increased MAR (+291%) and BFR/BS (+375%) at the early time point compared with BTX. In addition, at the late study time point, MS/BS (+167 and +397%), MAR, and BFR/BS were increased to a level significantly above Ctrl in animals treated with either PTH or ABL, respectively. At the femoral mid-diaphyseal endocortical surface, the effect of PTH and ABL did not differ, except for MS/BS (+86%) and BFR/BS (+98%), where ABL had a significantly more pronounced effect (**Table 5**).

**Table 5:** Dynamic bone histomorphometry at the cortical femoral mid-diaphysis of rats with the right hindlimb immobilized with BTX and treated with PTH or ABL.

	Base	Ctrl	BTX	BTX + PTH	BTX + ABL
Endosteal bone surface (week 0-1)					
MS/BS (%)		11.2 ± 8.14	11.4 ± 10.3	31.1 ± 18.1^a,b^	30.0 ± 14.1^a,b^
MAR (μm/d)		1.21 ± 1.57	0.55 ± 1.29	2.15 ± 1.25^b^	1.43 ± 1.09
BFR/BS (μm^3^/μm^2^/d)		0.22 ± 0.30	0.16 ± 0.41	0.76 ± 0.55^a,b^	0.50 ± 0.39
Endosteal bone surface (week 2-3)					
MS/BS (%)		8.60 ± 21.03	8.36 ± 14.6	23.0 ± 9.39^a,b^	42.7 ± 10.3^a,b,c^
MAR (μm/d)		0.00 ± 0.00	0.61 ± 0.94	1.17 ± 0.82^a,b^	2.10 ± 0.45^a,b^
BFR/BS (μm^3^/μm^2^/d)		0.00 ± 0.00	0.16 ± 0.31	0.46 ± 0.32^a,b^	0.91 ± 0.35^a,b,c^
Periosteal bone surface (week 0-1)					
MS/BS (%)		67.1 ± 16.5	24.2 ± 16.6^a^	28.0 ± 20.8^a^	26.0 ± 8.7^a^
MAR (μm/d)		1.98 ± 0.18	1.53 ± 0.55	2.18 ± 0.50	2.03 ± 0.36
BFR/BS (μm^3^/μm^2^/d)		1.33 ± 0.36	0.39 ± 0.34^a^	0.57 ± 0.41	0.52 ± 0.18
Periosteal bone surface (weeks 2–3)					
MS/BS (%)		84.2 ± 11.3	55.3 ± 16.3^a^	54.6 ± 17.6^a^	52.6 ± 13.27^a^
MAR (μm/d)		3.63 ± 0.52	3.78 ± 0.66	4.42 ± 0.80	4.06 ± 0.72
BFR/BS (μm^3^/μm^2^/d)		3.05 ± 0.58	2.12 ± 0.80^a^	2.37 ± 0.93^a^	2.18 ± 0.82^a^

Bone was double labeled with calcein at day two and day nine (early time point: weeks 0−1) and double labeled with alizarin at day 16 and day 23 (late time point: weeks 2−3). MS/BS, mineralizing surface; MAR, mineral apposition rate; BFR/BS, bone formation rate. Data are presented as mean ± SD. ^a^p < 0.05 vs. Ctrl. ^b^p < 0.05 vs. BTX. ^c^p < 0.05 vs. BTX + PTH.

#### Femoral Mid-Diaphysis: Periosteal Surface

Disuse significantly reduced MS/BS (−64 and −34%) and BFR/BS (−71 and −30%) at the early and late time point, respectively. Neither PTH nor ABL were able to counteract the immobilization-induced decrease in MS/BS at the early time point and MS/BS and BFR/BS at the late time point. Finally, no significant difference in animals treated with ABL compared with PTH were seen at the femoral mid-diaphyseal periosteal surface (**Table 5**).

#### Proximal Tibial Metaphysis: Trabecular Bone

**Figure 8:**
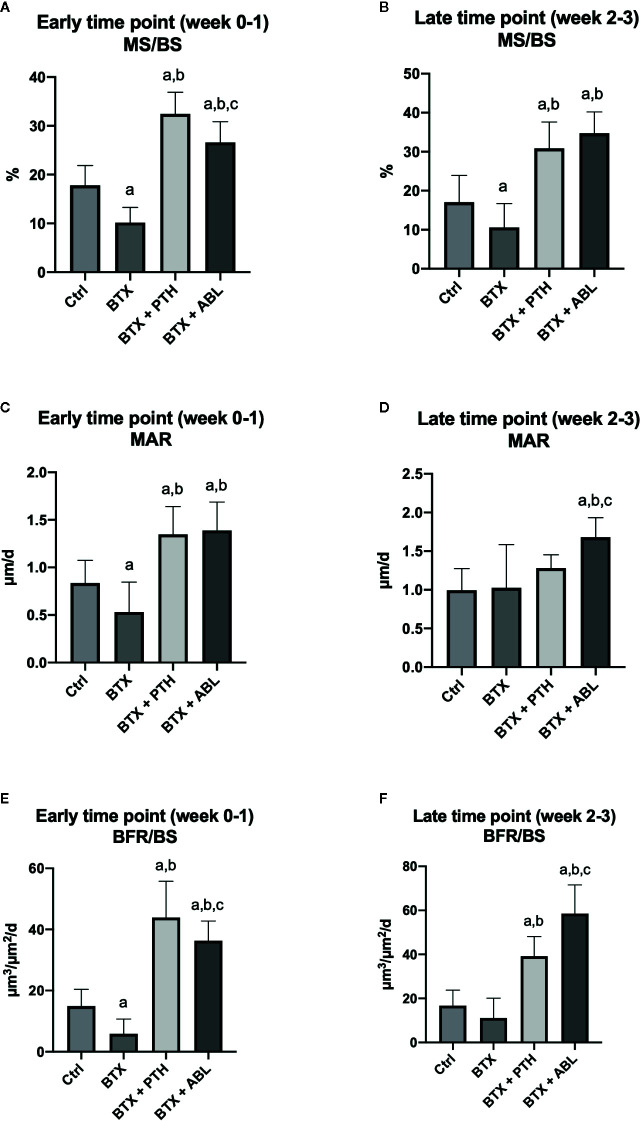
Dynamic bone histomorphometry on the proximal tibial metaphysis of rats with the right hindlimb immobilized with BTX and treated with PTH or ABL. **(A, B)** MS/BS, mineralizing surface; **(C, D)** MAR, mineral apposition rate; **(E, F)** BFR/BS, bone formation rate. Data are presented as mean ± SD. a: p < 0.05 vs. Ctrl. b: p < 0.05 vs. BTX. c: p < 0.05 vs. BTX + PTH.

BTX significantly reduced MS/BS (−43%), MAR (−37%), and BFR/BS (−60%) at the early time point and decreased MS/BS (−38%) at the late time point compared with Ctrl. Both PTH and ABL completely prevented the disuse-induced reduction of MS/BS (+83 and +49%), MAR (+61 and +65%), and BFR/BS (+193 and +140%) at the early time point and MS/BS (+82 and 104%) at the late time point compared with Ctrl, respectively. PTH significantly increased MS/BS (+22%) and BFR/BS (+22%) compared with ABL at the early time point. At the late time point, the opposite was the case, where ABL significantly increased MS/BS (+12%) and BFR/BS (+51%) compared with PTH (**Figure 8**).

Immobilization significantly increased Oc.S/BS (+114%) compared with Ctrl. Treatment with PTH significantly decreased Oc.S/BS (−45%) compared with BTX, while Oc.S/BS did not differ between ABL treated animals and BTX animals. Treatment with ABL significantly increased OS/BS (+176%) compared with BTX, while the increase in OS/BS (+95%) induced by PTH was not enough to elevate OS/BS to a level statically above that of BTX. Finally, no significant difference in Ob.S/BS was found between any of the groups compared (**Table 6**).

**Table 6:** Osteoblast surface and osteoclast surface at the proximal tibial metaphysis of rats with the right hindlimb immobilized with BTX and treated with PTH or ABL.

	Base	Ctrl	BTX	BTX + PTH	BTX + ABL
Proximal tibial metaphysis					
OS/BS (%)	2.83 ± 2.22	2.00 ± 1.47	1.60 ± 1.36	3.12 ± 2.25	4.41 ± 3.05^b^
Ob.S/BS (%)	3.21 ± 2.04	1.57 ± 1.09	1.49 ± 1.34	2.44 ± 2.17	3.26 ± 3.03
Oc.S/BS (%)	12.4 ± 5.04	6.50 ± 4.52	13.9 ± 4.57^a^	7.60 ± 3.24^b^	10.1 ± 4.37

OS/BS, osteoid covered surfaces Ob.S/BS, osteoclast covered surfaces; Oc.S/BS, osteoclast covered surfaces. Data are presented as mean ± SD. ^a^p < 0.05 vs. Ctrl. ^b^p < 0.05 vs. BTX.

## Discussion

The purpose of the present study was to compare on a mole-to-mole basis the ability of the bone anabolic peptides PTH and ABL to prevent immobilization-induced bone loss in rats. The main finding of the study was that treatment with PTH or ABL was in general equally effective in preventing loss of bone mass, deterioration of trabecular microarchitecture, and bone strength in hindlimb-immobilized rats. However, minor site-specific differences between the effects of PTH and ABL were observed.

As expected, injection of BTX into the hindlimb musculature resulted in a substantial reduction in gait ability, muscle mass, bone mass, bone strength, and a deterioration of the trabecular microarchitecture. These findings underline the well-established rapid and pervasive impact of relatively short-term immobilization on bone tissue and are in accordance with previous studies of BTX-immobilized mice (31, 34–36) and rats (12, 37–39).

Treatment with PTH counteracted the immobilization-induced reduction of femoral aBMD and BMC, femoral metaphyseal and epiphyseal and vertebral BV/TV and Tb.Th as well as femoral neck strength. In addition, PTH increased MS/BS, MAR, and BFR/BS compared with BTX at the tibial metaphysis at both the early (weeks 0–1) and late time point (weeks 2–3). The immobilization-induced bone loss at the proximal tibial metaphysis seemed to be inhibited by PTH since Oc.S/BS was reduced to a level similar to that of control animals. This inhibitory effect on osteoclast covered surfaces is in agreement with several previous studies by us (28, 40) and others (41). Our findings confirm that PTH can prevent immobilization-induced bone loss in rats and are in accordance with previous studies (12, 28, 40).

ABL prevented the disuse-induced decrease in femoral aBMD and BMC, femoral metaphyseal and epiphyseal Tb.Th, and epiphyseal BV/TV. Furthermore, tibial metaphyseal MS/BS, MAR, BFR/BS were increased compared with BTX at both the early (week 0-1) and late time point (week 2-3). However, in contrast to PTH, ABL did not completely reverse the immobilization-induced loss of femoral metaphyseal BV/TV, although the effect of PTH and ABL did not differ significantly. It may seem surprising that treatment with PTH resulted in a decreased Oc.S/BS, when treatment with ABL did not. However, the difference in Oc.S/BS between BTX and BTX + ABL tended toward statistical significance (*p* = 0.10). Furthermore, ABL was not able to prevent the BTX-induced loss of femoral neck strength, while this was the case for PTH. However, the difference in femoral neck strength between BTX and BTX + PTH was just slightly below the level of significances (*p* = 0.03). Therefore, it cannot be ruled out that the experiment had been repeated or if even more animals were included that the outcome would have been different.

In contrast to treatment with PTH, no studies have previously investigated the effect of ABL in relation to disuse-induced bone loss in rats. However, ABL has previously been studied in intact (42, 43), ovariectomized (44, 45), and orchiectomized animals (46, 47), as well as during fracture healing in mice (26) and rats (48). These studies have demonstrated bone anabolic properties of ABL in accordance with those established in the present study.

In the present study, the efficacy of PTH or ABL revealed only few differences. These differences were only observed in isolated skeletal sites and any significant difference between PTH and ABL were confined to tissue mineral density, structure model index, and dynamic bone histomorphometry. The overall similar effect of PTH and ABL on immobilization-induced bone loss is not surprising since they act through the same target receptor, PTH1R, which is expressed on osteoblasts (49). The few site-specific differences between PTH and ABL might be explained by differences in receptor conformation selectivity, since PTH preferentially binds to the R^0^-conformation, while ABL is selective toward the R^G^-conformation of PTH1R (19, 20).

In the present study, neither treatment with PTH nor ABL could inhibit the BTX-induced loss of Tb.N. The reason for this may be that the effect of PTH is bone anabolic and not antiresorptive. Indeed, PTH increase both bone resorption and bone formation, but the bone formation outweigh the bone resorption resulting in a net bone anabolic effect (50). Although, it has been reported that ABL increase bone formation in ovariectomized rats without increasing bone resorption (51) this view has been challenged (52). Moreover, a large clinical study of osteoporotic postmenopausal women has shown that treatment with ABL result in increased markers of both bone formation and resorption (22). This is consistent with the present study, which found that PTH and ABL lead to similar amounts of osteoclast covered surfaces at the proximal tibial metaphysis. This finding is further corroborated by a very recent study, which showed that the same dose of PTH and ABL resulted in similar serum CTX values in intact female mice (43). Therefore, despite the overall anabolic effect of PTH and ABL, the elevated bone turnover increases the likelihood of perforating trabeculae. It is generally accepted that the loss of trabeculae is irreversible, and therefore lost trabeculae cannot be replenished by a bone anabolic therapy. However, that does not mean that the bone volume fraction cannot be rescued by a bone anabolic treatment as long as trabeculae are still in place as a scaffold for the osteoblasts to form bone. We and others have previously reported that treatment with PTH was unable to prevent loss of Tb.N in the BTX-disuse model (12, 28, 53).

In a murine fracture model, Bernhardsson et al. showed that ABL and PTH can stimulate bone regeneration to a similar extend (26). A recent study by Le Henaff et al. have demonstrated that ABL and PTH at the same dose has the same effect on bone tissue in intact mice (42). Another recent report from Sahbani et al. also showed similar effect of ABL and PTH at doses comparable to those used in the present investigation (43). In contrast to these pre-clinical findings, clinical studies have reported greater aBMD response rates in osteoporotic patients treated with ABL compared with patients treated with PTH (23, 54). However, in these clinical studies, the dosage of ABL was up to four times higher than the dosage of PTH. The strength of the present study is that the amount of PTH and ABL administered is directly comparable, as the two agents are given in exactly the same molar concentration.

Perhaps surprisingly, the serum calcium concentrations did not differ between animals treated with PTH and ABL. The clinical study by Miller et al. demonstrated that daily treatment with 80 µg of ABL lead to a lower incidence of hypercalcemia (3.4%) than daily treatment with 20 µg PTH (6.4%) (22). However, Arlt et al. compared the bone resorption marker CTX-I in mice treated with PTH and ABL at a dose of 10 µg/kg/day and found that the CTX-I values did not differ between the two treatments (55). These findings are cooperated by the study of Le Henaff et al., which also showed no differences in serum CTX-I in mice treated with PTH and ABL at a daily dose of 80 µg/kg. These bone resorption markers are consistent with the serum calcium findings of the present study. The direct serum calcium measurements in rats of the present study is thus cooperated by previous inferred calcium levels made using resorption markers in mice. However, the observations that ABL and PTH leads to similar serum calcium concentrations in rodents is at variance with the clinical finding that treatment with ABL leads to a lower incidence of hypercalcemia than treatment with PTH (22, 23). The reason for the different outcome in the clinical and preclinical studies may be attributed to species differences between humans and rodents.

The present study highlights the efficacy of PTH in preventing immobilization induced bone loss (56) and for the first time establish that ABL has similar effect in counteracting the disuse induced degradation of the skeleton.

A limitation of the present study is the relatively high dosage of PTH compared to the dosage used in the clinic. However, the dosage was chosen in order to maximize the bone anabolic effect as previously described (12). In addition, the dosages of PTH and ABL are similar to those used in previous pre-clinical studies by Le Henaff et al. and Sahbani et al. (42, 43). It seems tempting to measure biochemical markers of bone formation or resorptions in serum. However, in the present study, we decided not to measure bone turnover markers since we have previously shown in BTX hindlimb immobilized animals that the markers are “diluted” by the part of the animal not undergoing immobilization (39).

Treatment with PTH or ABL, when given in the same molar concentration, have very similar efficacy in counteracting the immobilization-induced loss of bone mineral density, deterioration of trabecular microarchitecture, and reduction of bone strength in rats.

In conclusion, the study shows for the first time that PTH and ABL has very similar efficacy in preventing disuse osteopenia in immobilized rats when given in the same molar concentration.

## Data Availability Statement

The raw data supporting the conclusions of this article will be made available by the authors, without undue reservation.

## Ethics Statement

The animal study was reviewed and approved by The Danish Animal Experiment Inspectorate (2018-15-0201-01436).

## Author Contributions

Study design: MB, JT, and AB. Study conduct: MB and AB. Data collection, data analysis, and interpretation: MB, JT, and AB. Manuscript draft: MB. Figures and graphical design: MB. Manuscript revision: MB, JT, and AB. All authors contributed to the article and approved the submitted version.

## Conflict of Interest

The authors declare that the research was conducted in the absence of any commercial or financial relationships that could be construed as a potential conflict of interest.
